# Radiotherapy commissioning and tariff—how can we deliver advanced, innovative, and personalised radiotherapy?

**DOI:** 10.1093/bjr/tqaf253

**Published:** 2025-10-21

**Authors:** Imogen Powell Brown, Daniel Hutton, Nicola Thorp, James Thomson, Ran MacKay, Liesl Hacker, Lisa Ashmore, John Hayes, John Archer, Carl Rowbottom

**Affiliations:** NW Radiotherapy Specialised Services Clinical Network, Manchester M20 4BX, United Kingdom; NW Radiotherapy Specialised Services Clinical Network, Manchester M20 4BX, United Kingdom; Lancaster University, Lancaster LA1 4YW, United Kingdom; The Christie, Manchester M20 4BX, United Kingdom; The Clatterbridge Cancer Centre, Liverpool L7 8YA, United Kingdom; The Christie, Manchester M20 4BX, United Kingdom; The Christie, Manchester M20 4BX, United Kingdom; Lancaster University, Lancaster LA1 4YW, United Kingdom; Cheshire and Merseyside Cancer Alliance, Liverpool L7 8YA, United Kingdom; The Christie, Manchester M20 4BX, United Kingdom; The Clatterbridge Cancer Centre, Liverpool L7 8YA, United Kingdom

**Keywords:** Radiotherapy funding, Radiotherapy tariff, Value Based Payment, Advanced radiotherapy, Innovative radiotherapy

## Abstract

Radiotherapy practices have changed significantly over recent decades with the introduction of increasingly personalised approaches to preparation and treatment and the use of a wider range of imaging technology, treatment techniques and software such as Artificial Intelligence (AI). The lack of development of the radiotherapy tariff, which remunerates services for radiotherapy delivery, has contributed to poor adoption rates and inequitable access for patients to new advanced treatment technologies and software across England. The radiotherapy tariff has a potential to be a lever to drive innovation across the system, if it is routinely updated to respond to latest clinical consensus. The commissioning of radiotherapy services is being transferred from NHS England to Integrated Care Boards (ICBs), in their emerging role as ‘strategic commissioners’. Along with wider reform to funding mechanisms set out in the 10 Year Health Plan, this presents an opportunity to reshape the commissioning and tariff structures for radiotherapy services to better reflect contemporary radiotherapy practice. This paper explores the limitations of current funding arrangements for radiotherapy. It proposes recommendations to ensure that providers are supported to deliver more productive, innovative and value-based radiotherapy.

This paper is part of a series of three papers, on (1) radiotherapy tariff, (2) radiotherapy capital spending and (3) holistic aspects of radiotherapy funding, which together consider what a sustainable, innovative and person centred radiotherapy funding model looks like as specialised services commissioning is delegated to Integrated Care Boards.

## Background

Radiotherapy is an integral part of treatment provision for cancer patients in England and is utilised in around 40% of cancer survival cases.^1^ Despite its important role within cancer care, radiotherapy takes up a small proportion of the cancer budget at around 7%, making it highly cost effective.^2^ The potentials to improve efficiencies within radiotherapy funding further are continually expanding, largely thanks to advances in technology.^3^

In recent decades radiotherapy has become more personalised and accurate with greater advancements in pre-treatment imaging techniques and delivery of radiotherapy. This technological focus and research into radiotherapy delivery has contributed to improving patient outcomes, including survival and patient experience. It also opens avenues to more cost- effective and shorter treatment cycles for patients, which will be important as demand for cancer treatment increases.^4^ Progress is in line with UK Government and NHS England (NHSE) ambitions to make the health service more efficient, innovative and productive, and the NHS England Radiotherapy Service Specification which expects providers to deliver 'advanced and innovative’ care.^5^

Across Europe many reimbursement models for radiotherapy have struggled to evolve with advancements in clinical techniques or technology.^6^ In England, the current radiotherapy funding mechanisms were introduced as part of the Payment by Results (PbR) system over 10 years ago, which has since been used widely as a reimbursement model across the NHS. Radiotherapy was incorporated into the NHS Payment Scheme in 2023 with Healthcare Resource Groups (HRGs) or ‘tariff codes’ remaining the currency of the radiotherapy tariff by grouping clinically similar procedures based on resource use. In April 2024 some amendments were made to specialist radiotherapy HRGs (Stereotactic Radiosurgery/Radiotherapy, Selective Internal Radiation Therapy, Stereotactic Ablative Body Radiotherapy, Brachytherapy).^7^ However, overall, the tariff has not kept pace with the complexity of radiotherapy since its introduction which means funding arrangements do not contain the requisite sophistication or agility to sufficiently distinguish the scope of advanced and innovative radiotherapy services.^2^

The tariff, in its current format, has been intermittently updated to renumerate adopted practice rather than encourage and reward implementation of novel advanced radiotherapy techniques. This reactive nature of the radiotherapy tariff makes services slow to fund and implement latest innovations, such as AI auto contouring, hypofractionation and personalised treatment techniques, which is inhibiting efforts to modernise and drive efficiency within services. Combined with this, rising inflationary costs have left providers unable to keep up with the cost of radiotherapy, and as the majority of activity is now coded as the highest complexity there is effectively no differentiation nor financial incentive to innovate further. This reduces radiotherapy providers’ ability to invest in advanced treatment technologies and software, which could lead to inequitable access across England.^8^

## Advanced radiotherapy

A number of new technologies and techniques are now available to support advanced radiotherapy delivery, which are not adequately reflected within tariff arrangements.

Patients receiving radiotherapy require a pre-treatment scan to acquire a dataset to allow the accurate planning of treatment. This pre-treatment imaging is currently bundled into a single tariff code, assuming CT scan only use, which does not adequately capture the range and complexity of procedures used to personalise radiotherapy. For example, a significant proportion of patients now require contrast enhanced CT scans for radiotherapy treatment planning,^9^ whilst modern radiotherapy increasingly uses different imaging modalities such as PET/CT scans or MR scans along with CT scans.

AI auto-contouring software has becoming more routinely used within the radiotherapy pre-treatment pathway, supporting the transition from total manual delineation of organs for treatment planning to greater automation.^10^ This means that a time- intensive and resource heavy process, involving clinical oncologists, physicists, and radiographers, is avoided, enabling staff to focus on other critical activities and potentially reduce waiting times.^11^ Costs of AI software solutions in treatment planning are not reflected within the current tariff codes, which results in providers depending on various alternative, often short- term funding streams. Recent fixed term investment from Government has not materialised into the system, raising the risk that some providers will be unable to continue to utilise AI contouring software across patient pathways.^12^

The delivery of radiotherapy treatment has also become more complex. The current tariff structure combines patient immobilisation, patient monitoring, on-treatment verification imaging, treatment of a fraction of radiotherapy, and patient dose verification activities into a single HRG code. There is now much greater personalisation of treatment techniques for modern radiotherapy, including adaptive radiotherapy techniques, so that treatment plans can be updated as needed during a patient’s treatment cycle.

The original structure of the tariff system inhibited the adoption of certain innovations in the past decade and has lacked the flexibility to respond as new techniques and technologies emerge. Stereotactic Ablative Body Radiotherapy (SABR) is a hypofractionation technique that reduces the number of radiotherapy appointments a patient needs in their treatment cycle by increasing radiotherapy dosage per appointment. The innovative technique is linked to improved survival outcomes for certain patients^13^ alongside being more convenient for patients and a more efficient use of healthcare care resources.^14^ The tariff payment was set up to reimburse providers on the number of fractions which made the use of codes an inappropriate means of remunerating providers for hypofractionation.^15^ Particularly as SABR often requires more complex planning, quality assurance and imaging verification than conventional radiotherapy. NHS England introduced a payment structure for SABR for certain tumour sites in 2024/25. This is a pathway-based approach that pays for a course of treatment, including preparation and the fractions delivered for that course of treatment, rather than paying on a per fraction basis. This payment model was welcomed by radiotherapy providers but follows a number of years in which SABR was disincentivised by the tariff. Moving forwards, it is also unclear how more tumour sites will be added to this new payment structure where appropriate.

High levels of inflation globally and costs passed on by commercial manufacturers of radiotherapy equipment and peripherals has left HRG tariff prices out of pace. For a single radiotherapy machine, Linear Accelerator, delivering 7500 fractions of radiotherapy to 400 individual patients during a year, the payment received via the 2023/24 tariff is approximately £380 000 lower than if the tariffs had kept pace with inflation since 2014, using Consumer Price Index (CPI) inflation data^16^^,^^17^ (Table 1). This makes it difficult for radiotherapy providers to invest in modern radiotherapy technologies, software and techniques as commercial costs have increased in line with inflation.

**Table 1.** Tariff prices versus increased costs using CPI data. CPI is the measure used by the UK Government to set the Bank of England’s target for inflation.

Description	2014/15	2023/24	With inflation (CPI)	Difference
Preparation for simple radiotherapy with imaging and dosimetry	£379	£457	£508	−£51
Preparation for intensity modulated radiation therapy	£972	£1,001	£1,302	−£301
Preparation for intensity modulated radiation therapy, with technical support	£1,296	£1,318	£1,736	−£418
Preparation for complex conformal radiotherapy	£568	£675	£761	−£86
Preparation for complex conformal radiotherapy, with technical support	£757	£899	£1,014	−£115
Deliver a fraction of treatment on a megavoltage machine	£88	£102	£118	−£16
Deliver a fraction of complex treatment on a megavoltage machine	£121	£129	£162	−£33
Deliver a fraction of adaptive radiotherapy on a megavoltage machine	£178	£170	£238	−£68

## Creating a reactive tariff system

The commissioning of certain specialised services, including radiotherapy, are being delegated to Integrated Care Boards (ICBs) marking a significant shift in the NHS funding landscape. While NHSE retains ultimate responsibility for services, the delegation places greater responsibility on ICBs to act as the ‘strategic commissioner’ for its region to identify, resource and integrate innovative approaches, across a broader range of services and cancer treatments. However, ICBs will struggle to implement new innovations if inhibiting structures in funding arrangements disincentivise spending. Through reform introduced by NHS England—and continued within the Department of Health and Social Care (DHSC)—the radiotherapy tariff could be positioned as a proactive tool, responsive to innovation, evolving service specifications, and inflationary pressures.

The change would be in line with intentions set out in the UK Government’s 10- Year Health Plan to replace national tariffs based on average activity costs to an outcome-based payment system which encompasses best clinical practice and good quality care.^18^ It could represent a broader gradual shift towards more value-based payment approaches for certain services, as seen across Organisation for Economic Co-operation and Development (OECD) countries including cancer specific examples from France and the United States.^19^ Implementing this approach within radiotherapy funding would be consistent with recommendations from ESTRO HERO,^20^ including an analysis of radiotherapy reimbursement across Europe which suggests value- based funding models could be used to incentivise the adoption of innovation.^5^

**Figure 1.**
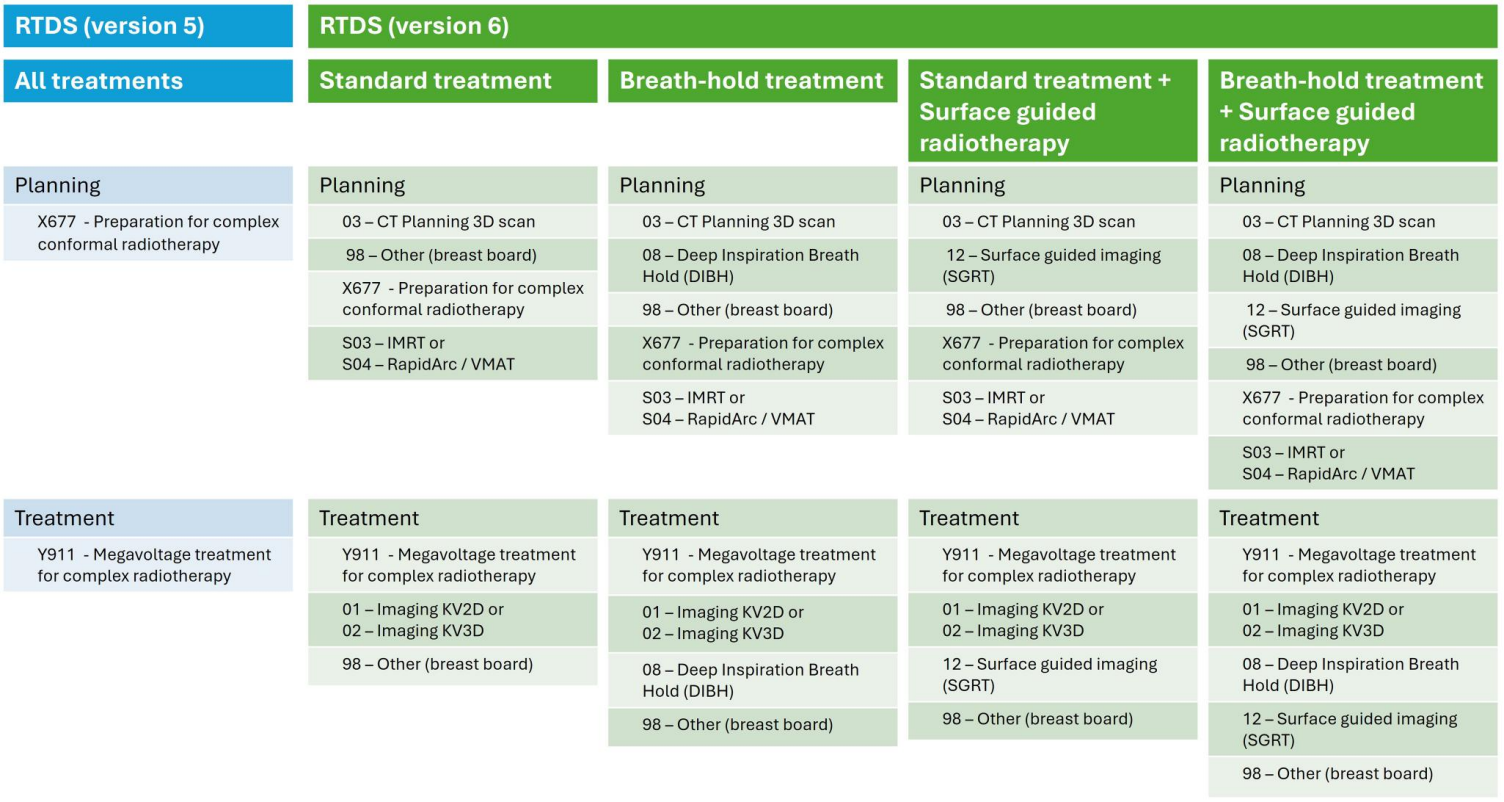
Overview of RTDS codes in Version 5 and Version 6 for breast cancer radiotherapy treatment.

There are already systems in place within radiotherapy that could inform value-based reimbursement approaches. Time driven activity-based costing could provide insight into real costs incurred during radiotherapy treatment.^5^ In England this could be informed by the Radiotherapy Data Set (RTDS). RTDS is a national standard for radiotherapy data across the NHS used to draw comparisons across radiotherapy providers on areas including pathways, prescription, planning and attendance. RTDS is informed by codes inputted by clinical teams, NHS England and DHSC could explore using the same codes as a reference for the tariff. This could make HRGs more responsive to real time operational activity and innovative techniques. Providers moving towards Version 6 of RTDS (Figure 1), which better reflects practice across pre-treatment and treatment activity, could start this process.

Amendments to tariff codes should also reflect latest clinical consensus and research. An independent national body, such as the Radiotherapy Board, could facilitate this by convening experts to review best practice and make recommendations for regular updates to the radiotherapy tariff. The Clinical Reference Group (CRG) model, already utilised by the National Programme of Care for Cancer within NHSE, provides an existing template of how the Radiotherapy Board could operate this new clinical advisory role to NHSE and DHSC as revision cycles for the tariff are put in place.

## Conclusion

The implementation of the 10 Year Health Plan, along with the National Cancer Plan for England, gives the Government renewed focus on what the long term and sustainable funding model for radiotherapy should look like, encompassing broader funding areas such as capital spending. As part of this, the NHSE and DHSC should redefine the commissioning and tariff for radiotherapy services, so that as national delegation of specialised services to ICBs goes ahead, commissioners can support the development and adoption of modern and outcome focussed radiotherapy.

This will require comprehensive tariff amendments to reflect current clinical best practice, complexity, nuance and costs, with a broader range of HRGs to reflect expansions in pre-treatment scans and radiotherapy techniques. Furthermore, in an era of rapid technological development and inflationary costs it is important that a new mechanism is put in place nationally to regularly review the reformed coding, commissioning, and tariff arrangements in line with operational activity and clinical consensus.
